# Limitations and opportunities of whole blood bilirubin measurements by GEM premier 4000®

**DOI:** 10.1186/s12887-017-0842-8

**Published:** 2017-03-29

**Authors:** Li Wang, Arianne Y. K. Albert, Benjamin Jung, Keyvan Hadad, Martha E. Lyon, Melanie Basso

**Affiliations:** 1 BC Children’s & Women’s Hospital, University of British Columbia, 4500 Oak Street, Room 2J9, Vancouver, BC V6H 3 N1 Canada; 2 Women’s Health Research Institute, Children’s and Women’s Health Centre of British Columbia, University of British Columbia, Vancouver, Canada; 3 Department of Pediatrics, Children’s and Women’s Health Centre of British Columbia, University of British Columbia, Vancouver, Canada; 4 Department of Pathology and Laboratory Medicine, Royal University Hospital, Saskatoon Health Region, University of Saskatchewan, Saskatoon, Canada; 5 Perinatal Health Program, Department of Obstetrics and Gynecology, Children’s and Women’s Health Centre of British Columbia, University of British Columbia, Vancouver, Canada; 6 BC Children’s Hospital Research Institute, University of British Columbia, Vancouver, BC Canada

**Keywords:** Neonatal, Hyperbilirubinemia, Screening, Whole blood bilirubin

## Abstract

**Background:**

Neonatal hyperbilirubinemia has traditionally been screened by either total serum bilirubin or transcutaneous bilirubin. Whole blood bilirubin (TwB) by the GEM Premier 4000® blood gas analyzer (GEM) is a relatively new technology and it provides fast bilirubin results with a small sample volume and can measure co-oximetry and other analytes. Our clinical study was to evaluate the reliability of TwB measured by the GEM and identify analytical and clinical factors that may contribute to possible bias.

**Methods:**

440 consecutive healthy newborn samples that had plasma bilirubin ordered for neonatal hyperbilirubinemia screening were included. TwB was first measured using the GEM, after which the remainder of the blood was spun and plasma neonatal bilirubin was measured using the VITROS 5600® (VITROS).

**Results:**

62 samples (14%) were excluded from analysis due to failure in obtaining GEM results. Passing-Bablok regression suggested that the GEM results were negatively biased at low concentrations of bilirubin and positively biased at higher concentrations relative to the VITROS results (*y* = 1.43x-61.13). Bland-Altman plots showed an overall negative bias of the GEM bilirubin with a wide range of differences compared to VITROS. Both hemoglobin concentration and hemolysis affected the accuracy of the GEM results. Clinically, male infants had higher mean bilirubin levels, and infants delivered by caesarean section had lower hemoglobin levels. When comparing the number of results below the 40th percentile and above the 95th percentile cut-offs in the Bhutani nomogram which would trigger discharge or treatment, GEM bilirubin exhibited poor sensitivity and poor specificity in contrast to VITROS bilirubin.

**Conclusions:**

An imperfect correlation was observed between whole blood bilirubin measured on the GEM4000® and plasma bilirubin on the VITROS 5600®. The contributors to the observed differences between the two instruments were specimen hemolysis and the accuracy of hemoglobin measurements, the latter of which affects the calculation of plasma-equivalent bilirubin. Additionally, the lack of standardization of total bilirubin calibration particularly in newborn specimens, may also account for some of the disagreement in results.

## Background

Jaundice is the most common condition that requires medical attention in newborns. Although it is benign and transient in most cases, some neonates with severe hyperbilirubinemia are at risk for bilirubin-induced neurologic dysfunction (BIND), which occurs when unconjugated bilirubin crosses the blood-brain barrier and binds to brain tissue causing irreversible neurologic damage if left untreated [1]. Prevention of BIND requires appropriate clinical assessment for risk factors, timely measurement of bilirubin levels and early treatment with either phototherapy or exchange transfusion [2]. Universal screening for newborn hyperbilirubinemia by measuring either total serum bilirubin (TSB) or transcutaneous bilirubin (TcB) has been adopted by many countries [3]. TSB has been the clinical standard for determining risk for kernicterus since the publication of Bhutani (1999) [4]. TcB is known to be less accurate at high bilirubin levels and may be affected by skin pigmentation (although improved with newer models) and skin thickening, making it unsuitable for older neonates [5, 6]. Most importantly, TcB is a physiologically different parameter than TSB because TcB assesses mainly extravascular bilirubin whereas TSB reflects the intravascular bilirubin concentration [6].

Whole blood bilirubin (TwB) analyzed on a blood gas instrument is a new and promising alternate method for neonatal hyperbilirubinemia screening. Compared to TSB it requires a smaller sample volume, has a faster turnaround time, and offers concurrent measurement of a full range of analytes (blood gas, electrolytes, glucose, lactate and co-oximetry), which allows for an efficient and comprehensive assessment of newborn status especially in critically ill neonates. Given these potential advantages of TwB, we conducted a prospective clinical study to compare TwB to TSB in the laboratory. Our objectives were to compare TwB results on the GEM Premier 4000® blood gas analyzer (Instrumentation Laboratory, Bedford, MA) (GEM) against plasma neonatal bilirubin results on the VITROS 5600® (Ortho Clinical Diagnostics, Rochester, NY) (VITROS) and to examine whether bilirubin measurement is influenced by various pre-analytical, analytical or clinical factors.

## Methods

### Patients

Three hundred and thirty-one newborns for whom bilirubin testing was clinically required were enrolled between May and August 2014 (total of 440 samples as some neonates had multiple samples). The inclusion criteria were for the neonates to be <14 days postnatal age and that sufficient blood volume was collected to enable measurement of both whole blood and plasma bilirubin. Capillary whole blood samples were collected into plain heparinized microtainers. TwB was measured using the GEM after which the remainder of the specimen was centrifuged and the clinically required plasma neonatal bilirubin was measured using the unconjugated and conjugated bilirubin (BuBc) slide on the VITROS. Clinical data such as gestational age, gender, birth weight, postnatal age, Apgar score and delivery methods (caesarean section (CS) vs vaginal delivery) were extracted from chart reviews. This study was approved by the Research Ethics Committee of the University of British Columbia (H13-02615). Consent was not required as this was a quality assessment project in the lab and no additional blood was drawn.

### Laboratory analysis principles

TwB measured by the GEM is a point-of-care test that was approved by the Federal Drug Administration in 2010. The blood gas analyzer measures total bilirubin along with the hemoglobin (Hb) fractions in hemolyzed whole blood samples by direct spectrophotometry in the co-oximetry module. In order to distinguish each component, multi-wavelengths are used and multi-variate regression algorithms established in a spectral library are compared when measuring a whole blood sample. Plasma equivalent bilirubin results are then reported using a formula [Bili plasma = bili whole blood/(1-hematocrit)], and hematocrit (Hct) is calculated as [Hct = 0.03x Hb]. The constant 0.03 represents the average concentration of hemoglobin (g/dL) within the red blood cells. GEM bilirubin is calibrated based on total bilirubin spectrum made of known levels of conjugated and unconjugated bilirubin. Since the GEM uses a direct spectrophotometric assay, it cannot distinguish different fractions of bilirubin.

Neonatal bilirubin concentrations in heparinized plasma were measured using the multi-layered slide technique by reflectance spectrophotometry on a VITROS 5600® analyzer. In the BuBc slide, plasma distributes and reacts with reagents while Hb and other interfering compounds are trapped and masked from being measured. Bu and Bc are measured simultaneously. The VITROS calibrator is composed of high grades of Bu that is traceable to NIST 916a and the synthetic ditaurobilirubin disodium salt for Bc. The Hemolysis index (H index) is a quantitative estimate of Hb that can be measured spectrophotometrically using the VITROS and was used to assess the effect of hemolysis on the measurement of whole blood bilirubin by the GEM.

### Statistical analysis

The study population was characterized by descriptive statistics. Passing-Bablok regression, Bland-Altman plots [7], and paired sample *t*-tests were used to examine the differences between the whole blood bilirubin (GEM) and BuBc measurements (VITROS). To determine the relationship between the difference in measurements by the GEM and the VITROS and clinical factors such as gestational age, gender, birth weight, Apgar score and delivery mode, we used Welch’s t-tests for comparisons between two groups, ANOVA for comparisons among more than two groups, and linear regression for relationships with continuous variables. Apgar scores were dichotomized into <7 and ≥7 groups for comparison. All comparisons of the above clinical variables were conducted on the first sample from each neonate.

We used generalized additive modeling to describe the association between the difference in methods and Hb concentration (as the relationship was clearly non-linear) and linear modeling to describe the association between the difference in methods and hemolysis (H index) on all samples, including the repeat samples on some neonates.

Finally, we assessed the predictive value of the GEM whole blood bilirubin results by applying the Bhutani nomogram [4] to classify them into the different risk categories. We also classified the VITROS values against the nomogram and used the numbers in each of the risk categories as the standard against which to compare the GEM results. We calculated sensitivity, specificity, negative predictive value (NPV), and positive predictive value (PPV) for risk cutoffs at the <40th percentile and >95th percentile, which would trigger discharge and treatment, respectively. Analyses were carried out using R version 3.2.4.

## Results

### Demographic

Table 1 shows the demographic and clinical variables of the study population. There were a total of 440 samples (from 331 newborns) collected during the study period. Three hundred and seventy eight samples (86%) were included in the analysis while 62 samples (14%) were excluded because the instrument reported whole blood bilirubin and/or co-oximetry results as incalculable. Among these 378 samples (from 318 neonates), 60 neonates had multiple bilirubin samples collected. The mean gestational age was 38.8 weeks and median postnatal age was 48 h. In total, 41% of neonates were delivered by CS while 59% were vaginal deliveries. Fifty-three percent of the study population was male.

**Table 1:** Demographic and clinical variables of the 318 unique neonates

Variable	
Gestational age (weeks), mean (SD)	38.8 (2.0)
Postnatal age (hours), median (IQR)^a^	48 (24–72)
Male gender	167 [53%]
Mode of delivery	
CS	112 [41%]
Vaginal	158 [59%]
APGAR at 1 min <7	21 [8%]
median (IQR)	9 (8–9)
APGAR at 5 min <7	10 [4%]
median (IQR)	9 (9–9)

^a^For all samples

### Passing-bablok regression

**Fig. 1:**
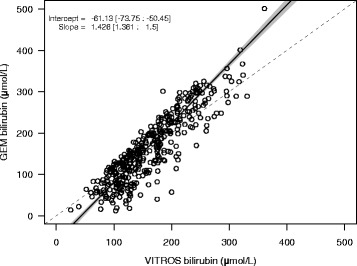
Comparison of results by Passing-Bablok regression. The *solid line* indicates the unbiased estimates of the intercept and slope from the regression. The *grey* indicates the 95%CI around those estimates. The *dashed line* shows the 1:1 line

The Passing-Bablok linear regression estimated the systematic difference (intercept) and proportional difference (slope) between the GEM and the VITROS (Fig. 1). There was a significant underestimation of bilirubin concentration with the GEM method at low bilirubin concentrations, whereas at bilirubin concentrations greater than 142 umol/L, the GEM method tended to overestimate bilirubin concentrations relative to the VITROS method. The regression equation was: y = 1.43x-61.13 (95% CI: 1.36 to 1.50 for the slope, and −73.8 umol/L to −50.5 umol/L for the y-intercept).

### Bland-Altman plot

**Fig. 2:**
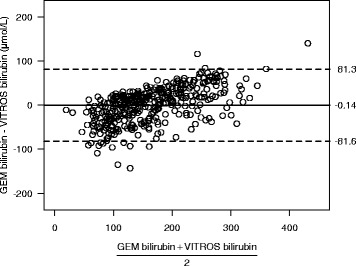
Bland-Altman plot. The *solid line* indicates the estimated bias and the *dashed lines* indicate the 95% limits of agreement

The Bland-Altman analysis estimated a mean bias of −0.14 umol/L (95% Limits of agreement = −81.6 umol/L to 81.3 umol/L) over the range of bilirubin concentrations tested. The minimum bilirubin was 25 umol/L, and the maximum bilirubin was 361 umol/L (Fig. 2). Although the mean difference (bias) is near zero, the GEM values underestimate at low concentrations of bilirubin and overestimate at higher concentrations, as revealed by the Passing-Bablok regression above.

### Hemoglobin effects/hemolysis effects

**Fig. 3:**
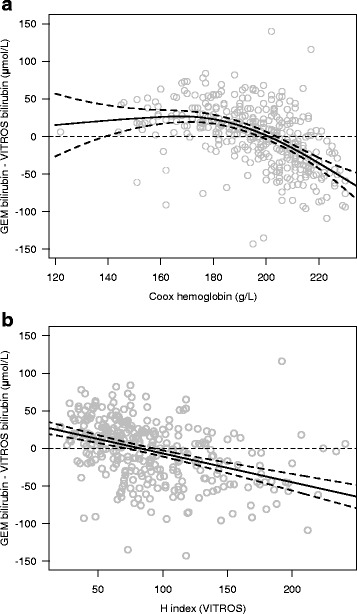
**a** The hemoglobin effect on the difference between GEM and VITROS: relationship between hemoglobin and the difference in bilirubin estimated by the two methods. The *solid line* indicates the additive model fit, and the *dashed lines* indicate the 95% confidence intervals. **b**. The hemolysis effect on the difference between GEM and VITROS: relationship between hemolysis (H index) and the difference in bilirubin estimated by the two methods. The *solid line* indicates the linear model fit, and the *dashed lines* indicate the 95% confidence intervals

The total Hb concentration in the study population ranged between 122 g/L to 230 g/L with a mean concentration of 195 g/L. No difference was detected in Hb concentrations between male (Mean: 195.8 g/L, SD = 19.8) and female infants (Mean = 195.3 g/L, SD = 18.3). Fig. 3a demonstrates the effect of Hb concentration on the measurement of whole blood bilirubin using the GEM method. The GEM bilirubin method tended to overestimate bilirubin when Hb was relatively low and underestimate it when Hb concentrations were high. The generalized additive model provided a much better fit to the data compared to a linear model (F test on change in deviance, *p* < 0.001) with an estimated number of degrees of freedom = 2.8 (*p* < 0.001). The model fit suggested that GEM tended to overestimate bilirubin concentration at Hb concentrations up to 200 g/L at which point it begins to strongly underestimate the bilirubin concentration. Fig. 3b demonstrates the effect of hemolysis on the measurement of whole blood bilirubin with the GEM method. Similar to Hb concentration, specimens with more hemolysis demonstrated a greater underestimation of bilirubin with the GEM methodology compared to the VITROS BuBc (slope = −0.4, *p* < 0.001).

### Clinical factors contributing to the differences in bilirubin measurement between the GEM and VITROS methods

A statistically significant difference in bilirubin levels was detected between male and female infants. When plasma bilirubin was measured using the BuBc slide, male infants had higher levels with an average concentration of 163.5 umol/L and standard deviation of 58.2 umol/L than female infants with an average of 143.4 umol/L and a standard deviation of 52.1 umol/L (Welch’s t = −3.2, df = 313, *p* = 0.001). A significant difference in whole blood bilirubin levels between male and female infants (Welch’s *t* = −3.2, df = 312.9, *p* = 0.002) was also detected when measured with the GEM methodology. Similar to the BuBc slide, male infants had higher whole blood bilirubin concentrations when compared to female infants (162.9 umol/L for male; 136.1 umol/L for female) when measured with the GEM method. Although method differences were observed in gender specific bilirubin levels, these differences were not statistically significant.

Similarly, we found that there were no significant effects of mode of delivery, gestational age, birth weight and Apgar scores on the difference between the two methods (data not shown). However, we found that there was a significant difference in the mean Hb concentrations in neonates delivered by CS in comparison to those delivered vaginally (Welch's t = −2.86, df = 230.1, *p* = 0.005). Babies born via CS had lower hemoglobin levels on average (190.8 g/L vs 197.7 g/L).

### Sensitivity, specificity, and predictive values for VITROS nomogram vs GEM nomogram

We examined the potential impact on clinical interpretation by comparing bilirubin results produced by the GEM versus the VITROS against the Bhutani nomogram. Five results from the dataset had to be excluded from this analysis because the time of collection was not known. For comparisons at the >95th percentile level, which would trigger treatment, the sensitivity (true positive rate) was 65%, specificity (true negative rate) was 84%, positive predictive value (PPV) was 46%, and the negative predictive value (NPV) was 92% (Table 2); while for comparisons at the <40th percentile which would trigger discharge, the sensitivity was 78%, specificity was 79%, PPV was 61%, and NPV was 90% (Table 3).

**Table 2:** GEM results of >95th percentile on the Bhutani nomogram compared to VITROS results of >95th percentile

		VITROS results		Totals
		Positive (treatment)	Negative (no treatment)	
GEM results	Positive (treatment)	42 (true positives)	50 (false positives)	92
	Negative (no treatment)	23 (false negatives)	258 (true negatives)	281
		65	308

**Table 3:** GEM results of <40th percentile on the Bhutani nomogram compared to VITROS results of >95th percentile

		VITROS results		Totals
		Positive (discharge)	Negative (no discharge)	
GEM results	Positive (discharge)	87 (true positives)	55 (false positives)	142
	Negative (no discharge)	24 (false negatives)	207 (true negatives)	231
		111	262	

## Discussion

This clinical study compared the bilirubin measurements between the whole blood GEM methodology and the plasma VITROS BuBc slide methodology in healthy newborns with capillary samples. We found an imperfect correlation between TwB and neonatal bilirubin such that bilirubin levels were underestimated at low concentrations by the GEM method while at concentrations greater than 142 umol/L, the GEM method tended to overestimate relative to the VITROS method. When classifying the risks for neonatal hyperbilirubinemia based on the Bhutani nomogram with these two methods, GEM exhibited low sensitivity and low positive predictive value in comparison to VITROS. Many factors including pre-analytical, analytical and clinical may have contributed to the observed differences between the methods.

First, although TwB is measured, the instrument reports plasma equivalent bilirubin using a calculation that relies on the measurement of Hb. While this conversion facilitates comparison with the Bhutani nomogram for risk stratification, inaccurate Hb leads to inaccurate plasma equivalent bilirubin results. Additionally, TwB was unreportable in 14% of samples because Hb was outside of the manufacturer’s claimed measurable range (30 g/L - 230 g/L). This observation could be related to the methodology used by the GEM to measure Hb or pre-analytical factors such as microclot formation due to poor sample mixing which may have affected the accuracy of the co-oximetry with falsely high Hb results. Interestingly, we did find that healthy term newborns within the first 2 days of life have higher normal Hb levels up to 239 g/L [8]. Neonatal Hb levels may be even higher due to polycythemia or other conditions and this will definitely challenge the analytical range of Hb by the GEM methodology (upper analytical range is 230 g/L). Specimens with Hb exceeding the 230 g/L will lead to a failure in generating a bilirubin result and a high failure rate would be problematic for a diagnostic device being considered as a screening tool. We also found that hemolysis can affect the difference between the GEM and the VITROS. Since all whole blood samples have to be hemolyzed before co-oximetry analysis, it is puzzling why hemolysis can affect accuracy. Based on the similar trends observed for both hemoglobin effect and hemolysis effect, we postulate this is just another reflection that high Hb concentration in the hemolyzed specimen can affect accuracy.

Second, the difference between results by the two methods might be related to differences in calibration. The GEM bilirubin is calibrated to total bilirubin, whereas the BuBc slides on the VITROS is calibrated to the respective fractions. Two tests are available from Ortho Diagnostics: neonatal bilirubin (BuBc) and total bilirubin (TBIL) [9], with BuBc being recommended for testing in neonates under 2 weeks of age and TBIL for patients 15 days old and above by the manufacturer [9]. The sum of Bu and Bc is not the same as total bilirubin because the latter contains not only Bu and Bc, but also delta bilirubin which is a bilirubin covalently bounded with albumin. Due to the lack of interference by Hb [10], the simultaneous measurement of Bu and Bc, and the absence of a clinically significant difference between BuBc and total bilirubin measurements in newborns (neonates have <2% delta bilirubin) [11, 12], we only offer BuBc in our hospital to avoid clinician confusion. Commercial calibrators used by field methods are known to affect the accuracy of bilirubin measurements on chemistry analyzers [13] and the clinical impact by different calibrators has been published recently. Kuzniewicz et al. [14] reported that the recalibration of the BuBc method by the manufacturer in 2012 led to a 39% relative reduction in infants with a TSB level of 257 umol/L and more than 50% reduction in both birth hospital phototherapy and readmissions for phototherapy. Similarly, the implementation of a new formulation of total bilirubin by another manufacturer (Roche diagnostics) also affected neonatal phototherapy rates even compared to its previous formulation [15]. Our comparison of GEM total bilirubin to BuBc instead of TBIL would add another challenge for the comparison with the whole blood bilirubin method by GEM. Standardization is needed for all bilirubin methods, which would improve the care for neonatal hyperbilirubinemia and allow for neonates to be monitored using different methods during the course of their disease.

We also analyzed possible contributing clinical factors to the observed differences in the methods and found a significant difference between methods for male and female infants. Additionally, we found that male infants had higher bilirubin levels than female infants in our patient population, which supports a previous report that male gender is associated with a higher risk of developing hyperbilirubinemia [16]. This suggests that the relationship between the difference in the methods and infant sex was at least partially driven by the relationship between average bilirubin levels and infant sex.

For comparisons at the >95th percentile level which would trigger treatment, sensitivity and PPV were 65 and 46%, respectively. These results suggest that had GEM bilirubin been used as the screening tool for risk stratification based on the Bhutani nomogram, 35% of neonates that would have been treated by VITROS results would not have been treated by the GEM results. Similarly, for low risk neonates (<40th percentile), who can be safely discharged, the sensitivity and PPV were 78 and 61%, respectively. This suggests that only 61% of those low-risk levels by GEM would be classified as low-risk by VITROS. These results support the conclusion that GEM bilirubin is neither sensitive nor specific enough to screen for neonatal hyperbilirubinemia.

Finally, we found that neonates delivered by CS had lower Hb concentrations than neonates delivered vaginally. This is consistent with the previous finding that neonates post vaginal deliveries have higher hemoglobin levels which could be due to the recommendation of delayed cord clamping which increases placental-fetal transfusion [17], whereas the duration of placental transfusion in CS tends to be shorter since an immediate cord clamping is often performed to avoid maternal bleeding, infections or other surgery-related complications [18]. A systematic review and meta-analysis study also indicated that CS compared with vaginal delivery, was associated with an increased placental residual blood volume which would cause a decreased level of hematological indices including hemoglobins in the neonates [19]. These interesting findings could be achieved in this study because of the availability of Hb measurements by whole blood gas analyzers. This is extremely useful when caring for babies with hyperbilirubinemia due to hemolytic causes as simultaneous availability of whole blood bilirubin and Hb results would reduce the total blood volume and time needed for both tests in the lab.

## Conclusions

This study suggests that TwB by the GEM is not yet ready for neonatal hyperbilirubinemia screening. Handling the difficult matrix of whole blood samples from newborns requires technological improvements to control pre-analytical and analytical variables. Ultimately, improvement of total bilirubin calibration with standardization and the accuracy of Hb measurements will help TwB become utilized clinically. With technical improvement, we believe whole blood bilirubin has the potential to become a valid screening tool considering the benefits of having additional analytes especially hemoglobin.

## Abbreviations

Bc: Conjugated bilirubin
Bu: Unconjugated bilirubin
CS: Caesarean section
GEM: GEM Premier 4000® blood gas analyzer
Hb: Hemoglobin
Hct: Hematocrit
TSB: Total serum bilirubin
TwB: Whole blood bilirubin
VITROS: VITROS 5600®
